# Approach to personalizing the treatment of osteoporosis

**DOI:** 10.1210/clinem/dgag257

**Published:** 2026-06-27

**Authors:** Gina Woods, Jennalee S Wooldridge, Connie M Weaver, Lora Giangregorio

**Affiliations:** Department of Medicine, University of California, San Diego, La Jolla, CA 92093, USA; VA San Diego Healthcare System, San Diego, CA 92161, USA; VA San Diego Healthcare System, San Diego, CA 92161, USA; Department of Psychiatry, University of California, San Diego, La Jolla, CA 92093, USA; School of Exercise and Nutrition Science, San Diego State University, San Diego, CA 92182, USA; Department of Kinesiology and Health Sciences, University of Waterloo, Waterloo, ON N2L 3G1, Canada

**Keywords:** osteoporosis, personalized medicine, shared decision-making, patient-centered care, exercise, nutrition

## Abstract

Despite the availability of multiple highly effective pharmacologic therapies for osteoporosis, fracture rates in the United States have plateaued or increased in recent years. At the same time, individuals with osteoporosis are exposed to an abundance of digital health information and frequently seek guidance on nutrition, exercise, and other lifestyle strategies to improve bone health. Many endocrinologists, however, may have limited time or expertise to address these questions comprehensively during routine clinical encounters. Current clinical practice guidelines provide valuable direction on selecting pharmacotherapy based on fracture risk, yet they offer limited guidance on how to personalize treatment plans by integrating patient values, beliefs, and preferences. The aim of this manuscript is to equip clinicians with practical, preference-sensitive strategies to address common concerns raised by postmenopausal women with osteoporosis, including evidence-based guidance on nutrition and on safe, effective resistance, impact, and balance-focused exercises for bone health and fall prevention. We also outline suggested approaches for responding to common concerns among patients who are reluctant to initiate pharmacologic therapy. Through 2 illustrative cases, we highlight how clinicians can integrate pharmacologic options with evidence-based lifestyle guidance and engage patients in shared decision making to develop individualized, goal-concordant care plans. Our goal is to provide clinicians with tools that support more effective, patient-centered conversations and ultimately improve the quality of osteoporosis care.

Osteoporotic fracture rates in the United States have plateaued or risen (eg, 22 to 25 per 1000 person-years among women ≥65 from 2013 to 2017), while treatment rates among high-risk patients declined from 9.2% to 7.4% between 2011 and 2022 (1-3). This widening gap highlights the need for more effective osteoporosis care delivery.

Postmenopausal women now have access to an unprecedented volume of digital health information, which may support more informed treatment decisions (4-6). Patients with osteoporosis frequently seek advice on nutrition, exercise, and other nonpharmacologic fracture-prevention strategies, yet endocrinologists may lack the time or expertise to address these issues comprehensively (7, 8). Patients bring diverse values and preferences that may not align with guideline-based osteoporosis treatment, and clinicians may be reluctant to deviate from established norms. These factors likely contribute to persistently low treatment rates: in 2022, only 50% of women with osteoporosis and 43% with fractures received therapy, underscoring the need for more personalized care.

This paper illustrates shared decision-making in osteoporosis care through 2 postmenopausal women with similar fracture risk but differing values and preferences. These cases demonstrate how individualized treatment plans can integrate clinical evidence, patient goals, and evidence-based nutrition, exercise, and pharmacologic strategies to support musculoskeletal health.

## Case 1

A 59-year-old woman presented to the endocrinology clinic for evaluation of osteoporosis. She underwent surgical menopause at age 43 for endometriosis and later developed moderate-to-severe vasomotor symptoms. When she asked about menopausal hormone therapy, her physician advised that it was not standard practice, and symptoms resolved by age 46. Her calcium intake included occasional yogurt and cheese but no milk. She consumed a balanced, omnivorous diet. She took supplemental calcium citrate 500 mg daily and vitamin D_3_ 600 IU daily. Her exercise routine included walking one mile on weekdays and hiking 2 to 3 miles on weekends.

She had no history of adult fractures and reported one non-injurious fall while hiking within the past year. Her initial DXA at age 59 showed osteoporosis with T-scores of −4.2 (lumbar spine), −2.6 (left total hip), and −2.4 (left femoral neck); TBS was 1.173 (degraded). Her primary care physician prescribed alendronate, but she developed abdominal pain and discontinued the medication after 4 doses.

On exam, she appeared healthy. Height was 5′7″, weight 160 lb (BMI 25 kg/m^2^), with normal gait and no kyphosis.

Evaluation for secondary causes of osteoporosis showed normal calcium, creatinine, phosphorous, 25-hydroxyvitamin D, parathyroid hormone, alkaline phosphatase, and serum protein electrophoresis; 24-hour urine calcium was 181 mg. Morning cortisol was 0.9 mcg/dL following 1 mg dexamethasone the prior evening.

She was diagnosed with osteoporosis (very high fracture risk) based on her lumbar spine T-score of −4.2, with early surgical menopause as her only clinical risk factor. She preferred a comprehensive and aggressive treatment approach incorporating nutrition, exercise, and pharmacologic therapy to maximally reduce fracture risk.

She was advised to consume 1200 mg of total daily calcium, emphasizing dietary sources first, and given a dietary protein target of 1-1.2 g/kg/day with varied food sources encouraged. In addition to walking, she was advised to engage in moderate to high intensity resistance training twice weekly with added impact exercise and balance training. Abaloparatide 80 mcg daily was initiated.

**Figure 1:**
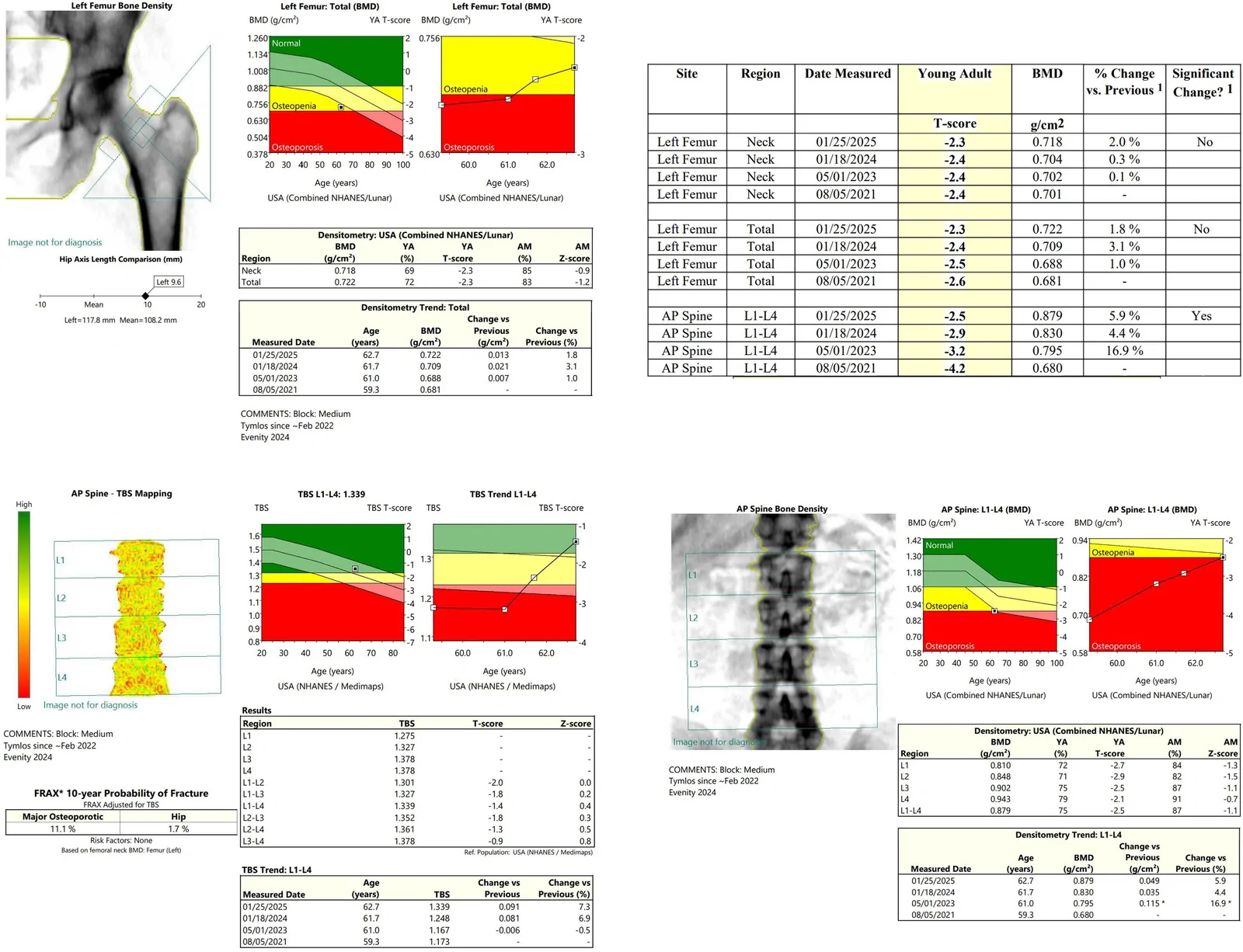
Sequential anabolic therapy (abaloparatide -> romosozumab) and associated gains in bone mineral density (case 1).

At two-year follow-up, she reported daily intake of yogurt and cheese with a 600 mg calcium carbonate supplement and protein from whole food sources at each meal. She was engaging in unsupervised resistance and impact training twice weekly, including jumping jacks with dumbbells, which led to a minor lower back strain. Adherence to abaloparatide was excellent. Repeat DXA showed a 21.3% increase in lumbar spine BMD (T-score −2.9) and a 4.1% increase in left total hip BMD (T-score −2.4). She was advised to only do impact exercise without dumbbells. Because of cost and her preference for at-home exercise, she was referred to a physical therapist for a few sessions to design a home program and review her form, with intermittent follow-up visits. Given persistent low BMD and her preference for maximal improvement, sequential anabolic therapy was selected to maximize gains in bone mineral density before transitioning to antiresorptive therapy. Romosozumab 210 mg monthly was initiated. One year later, DXA demonstrated an additional 5.9% increase in lumbar spine BMD (T-score −2.5) with normal TBS (1.339) and a left total hip T-score of −2.3 (Fig. 1). Following completion of romosozumab, she was transitioned to zoledronate 5 mg IV annually.

## Case 2

A 58-year-old woman presented to the endocrinology clinic for osteoporosis. She had no history of adult fractures. Her only medical condition was hypothyroidism, for which she was treated with levothyroxine.

She reported a long-term vegan eating pattern motivated by general health concerns. She took calcium and vitamin D3 supplements daily. For exercise, she attended OsteoStrong twice weekly and walked 3-4 miles daily. She reported no history of falls. Her last menstrual period was 4 years prior, and she continued to experience mild-to-moderate vasomotor symptoms.

On physical examination, she appeared well, with normal gait and posture. Her height was 5′3″, weight 162 lb, and BMI 28.7 kg/m^2^.

Laboratory results included normal calcium, phosphorous, creatinine, alkaline phosphatase, 25-hydroxyvitamin D, and TSH. DXA demonstrated a lumbar spine T-score of −2.0, left total hip T-score of −3.2, and left femoral neck T-score of −3.0. Trabecular bone score (TBS) was moderately degraded (1.253).

She strongly preferred to avoid pharmacologic therapy and was interested exclusively in nutrition and exercise interventions to support bone health. Because osteoporosis was her primary health concern and her vegan diet was motivated by perceived health benefits, she was advised that incorporating dairy and/or other animal protein sources may better support bone health. She was counseled to consume protein with each meal, from a variety of whole food sources. She was advised that the evidence on the effectiveness of Osteostrong is inconsistent and low quality, and there is limited safety information (9). She was referred to a small group, in-person resistance and impact training program supervised by an exercise physiologist. Considering her total hip T-score of −3.2, treatment with romosozumab was recommended, but was not authorized by her insurance and she declined any alternative pharmacotherapy.

One year later, she remained adherent to twice-weekly supervised resistance and impact exercise, continued calcium and vitamin D supplementation, and had added poultry and fish—but not dairy—to increase protein intake.

Repeat DXA showed a significant 4.3% increase in lumbar spine BMD (T-score −1.6) and a nonsignificant 4.0% increase at the left total hip (T-score −3.0) and left femoral neck (T-score −2.8).

After discussing bone health with peers in her exercise class, she inquired about menopausal hormone therapy (MHT) for vasomotor symptoms and skeletal protection. Despite some concerns about potential risks, she viewed MHT as a more natural approach that aligned with her healthcare preferences. In the absence of contraindications, transdermal estradiol and oral progesterone were initiated.

## Effective communication and behavior change

Consideration of patient's beliefs, values, and preferences is a cornerstone of evidence-based medicine (10), yet a review of English language clinical practice guidelines for osteoporosis found that less than half of guidelines reviewed (39%) mentioned patient beliefs, values, or preferences (11). We posit that anticipating, eliciting, and responding to these factors can facilitate more effective treatment plans.

## Discussing patient beliefs and preferences regarding pharmacological therapy for osteoporosis

Many patients hold beliefs or negative feelings about medications that impede treatment engagement. Patients have a range of concerns around medication taking relating to their personal preferences, past experiences, or misinformation (12). For example, beliefs that osteoporosis resulted from not exercising enough, wanting to delay treatment until it feels absolutely necessary, fearing jaw bone necrosis, or attributing a fracture to causes other than osteoporosis. Perceived benefit, trust in medications, symptomatic side-effects, safety, and perceived risk of fracture have all been shown to influence initiating or consistently using osteoporosis medications (13, 14). Consistent with temporal discounting, immediate side effects may be perceived as more important than the future benefit of fracture prevention, even in high-risk patients (15-17). Acknowledging these psychological barriers to medication use is important to developing collaborative patient-centered treatment plans. Understanding patients' specific concerns, normalizing them (“many people share these concerns”), and reinforcing that the decision is theirs can support shared decision-making.

## Motivational interviewing

Strategies from motivational interviewing can help to guide discussions about treatment recommendations, alternative options, and problem-solving barriers (18). Motivational interviewing is a collaborative, person-centered communication style designed to strengthen a person's own motivation and commitment to making behavior change. We describe a selection of motivational interviewing strategies that may be particularly amenable to osteoporosis care. These strategies can be used when discussing medication, nutrition, and exercise; however, examples pertaining to medication are provided here.

The OARS framework—using **o**pen-ended questions, **a**ffirmations, **r**eflective listening, and **s**ummaries—can be used to understand patient perspectives. Open-ended questions are intended to invite patients to share their perspective without being limited to a specific direction, eg, “What have you heard about bone health?” Affirmations are statements that recognize patients' strengths and efforts, eg, “You’ve done a lot of research on this, I can see the dedication you have to your health.” Reflective listening, is meant to foster trust and ensure understanding, eg, “It sounds like you’ve heard some really scary things about medications.” Summaries are similar to reflective listening and often pull together several points, eg, “It sounds like you really want to improve your bone density but are worried about the side-effects of medications. You’re not sure what to do. Did I leave anything out?” Providing information effectively can be done through asking permission (eg, “can I share some information about side-effects with you? Or “Is it OK if I share some of the concerns I have with that plan?”), and following an elicit-provide-elicit sequence (eg, “What do you know about osteoporosis?,” “The most effective way to increase bone density is through a combination of medication and health behavior change,” “What are your thoughts about that?”). A final motivational interviewing strategy that may be helpful is decisional balancing, or reviewing the pros and cons, from the patient's perspective, of 2 options. In osteoporosis care, this tool may help to highlight that not taking medications (or another behavior) also comes with risks. Table 1 shows common concerns and example responses using motivational interviewing and other strategies for effective communication.

**Table 1:** Common concerns and example responses using effective communication strategies

Common concern	Example response
I want to see if I can improve with more exercise.	It sounds like you’re motivated to improve your bone health. Exercise is a great way to improve musculoskeletal health. Most people require medication to raise their bone density out of the osteoporosis range (a T-score above −2.5) in the long-term, even with strong exercise and diet habits. I am happy to discuss both exercise and medication options today.
I’m concerned my calcium intake hasn’t been adequate. If I start taking calcium and vitamin D supplements now, will that be enough to improve my bone density?	Calcium and vitamin D are important, and low intake over time may contribute to osteoporosis. We take a comprehensive approach—making sure you’re getting adequate calcium and vitamin D, as well as enough protein, along with regular exercise and, when appropriate, medications to strengthen bone and reduce fracture risk.
I’m not ready to start medication. I want to wait until it is absolutely necessary.	I understand not wanting to take unnecessary medication and the choice to take medication is yours. Can I tell you the concerns I have with waiting? [If yes] Most of the time, bone density naturally declines with age, so delaying treatment can mean losing more bone and making it harder to move out of the osteoporosis range later. These medications work best when started early—when they can help preserve the bone you have and build on it—rather than trying to catch up after further loss. What are your thoughts about that?
I’ve heard these medications cause brittle bones.	This is common concern; some medications can cause rare thigh bone fractures after long-term use. We can help minimize the risk by limiting how long you stay on the medication. Alternative: It's tough when there are no completely risk-free options. Are you interested in reviewing the pros and cons of both options together?
My dentist told me not to take osteoporosis medication.	It's confusing to get different recommendations from different health care professionals. Your dentist may be thinking about a very rare side-effect, osteonecrosis of the jaw, that can occur following dental work. The chances of this already rare side-effect can be reduced with oral hygiene and having dental work completed prior to starting the medication. If you need dental work once you start treatment, I will work with you on the timing of the medications in relation to your dental procedures.
My fracture was not due to osteoporosis. Anyone would have had a fracture if they fell like I did.	A hard fall could certainly impact anyone. A tricky thing about osteoporosis is that it's often a silent condition. People usually don’t feel any different or know their bones are weaker until something like a fracture happens.

## Discussing nutrition and exercise beliefs and preferences

Comprehensive osteoporosis care extends beyond pharmacologic therapy to include nutrition and exercise counseling. As with medications, patients may hold values and preferences that require individualized recommendations. Because behavior change is more likely when goals are specific and actionable, tailored guidance is essential. For example, in Case 2, a patient following a vegan diet was counseled to ensure adequate protein intake through consistent consumption of diverse protein sources at each meal (19). A specific action plan for this patient might look like: “I will eat two different protein-rich foods (how much), three times a day (how often), at breakfast, lunch, and dinner (when).” For both diet and exercise behaviors, some patients may be motivated to make substantial changes, where others may benefit from making small, attainable, and gradual changes, even if initial goals are below recommended (20, 21). There is evidence that peer support and group-based physical activity programs can be effective for increasing physical activity (22, 23). In the context of osteoporosis, peer support could influence medication taking decisions and reduce medication hesitancy as in Case 2.

### Nutrition and bone health

For many clinicians, formal training in nutrition has been limited, and counseling for osteoporosis often focuses primarily on calcium and vitamin D, an emphasis that is also reflected in current clinical practice guidelines. While it is true that many patients do not meet recommended intake levels of these 2 essential nutrients, this represents a narrow view of nutrition. Bone is a living tissue, and its growth and maintenance depend on adequate intake of a full range of essential nutrients. Furthermore, aspects of the whole diet influence bone health and whole-body health. For example, energy intake influences adiposity and obesity. Fiber intake influences gut health. Protein and amino acid intake influences acid–base balance. Nutrition plays an important role in the most prevalent chronic diseases, including osteoporosis, but there is a great time lag before benefits or harms of dietary choices surface in these long latency diseases. Thus, good nutrition is important for prevention and rarely serves as a silver bullet to “cure” a disorder. Expert panels recommend food before supplements to achieve adequate nutrient intake (24). Patients should be counseled to choose a dietary pattern that meets their nutrient requirements, with an emphasis on nutrient-dense foods (Table 2) (25).

**Table 2:** Key nutrition talking points for osteoporosis care

Start with **food first**. Nutrient-dense foods give your body a full package of vitamins, minerals, protein, and other natural compounds that work together to support bone health.
Include calcium-rich foods daily. Aim for 1000-1200 mg of calcium per day, preferably from food. Three servings of dairy typically meet this goal without the need for supplements. For plant-based diets, calcium-fortified soy milk is a well-absorbed option, while other plant-based milks vary in calcium content and absorption has not been studied.
Prioritize high-quality protein at most meals. Protein is essential for bone strength—about half of bone is made of protein. What is “high-quality” protein? High-quality proteins contain all the building blocks your body needs (called essential amino acids) and are easy to absorb. These proteins are especially effective at supporting muscle and bone.
If you follow a vegetarian or vegan diet, you can still meet your protein needs for bone health. Plant-based foods often contain lower amounts of protein, so paying attention to your total protein intake is important. Plant proteins may not provide the full complement of essential amino acids, so combining different sources across the day helps ensure you’re getting everything your bones need. Aim for a mix of soy foods (tofu, tempeh, edamame), beans, lentils, nuts, seeds, whole grains, and—if you’re a lacto-ovo vegetarian—eggs and dairy.
Keep salt intake on the lower side. Too much salt can increase calcium loss from bones and raise blood pressure. Cooking at home and choosing less-processed foods helps.
Use supplements when food isn’t enough. If you’re low in calcium, vitamin D, or other nutrients, supplements can help fill the gap—but they work best alongside a healthy diet, not instead of one. Remember that supplements don’t provide fiber.
Fiber from plant foods supports a healthier gut microbiome, which plays a role in overall health—including bone health.

Patients are bombarded with marketing claims about dietary supplements and bone health, largely without evidence for benefits or risks. Dietary supplements are recommended when patients are unable to meet their nutrient requirements through food alone. Supplements generally deliver one or a limited number of nutrients, in contrast to whole foods, which provide a diverse matrix of vitamins, minerals, and bioactive compounds that may contribute to bone health. A recent review concluded that few micronutrients or bioactive compounds have evidence comparable to calcium (particularly from dairy) and exercise for supporting bone health (26). Unlike whole foods, dietary supplements lack the food matrix and dietary fiber that promote calcium absorption and gut health through fermentation and production of short-chain fatty acids (27).

The standard of care for nutritional advice for patients with osteoporosis has been to take calcium and vitamin D supplements. If the patient avoids dairy products that are rich in calcium and vitamin D (not fortified in all countries), supplementation is likely to be necessary to meet the recommendations for the general population of 1000 mg calcium/day for men aged 51-70 years or 1200 mg calcium/day for men over 70 years and women over age 50 years and 15 ug (600 IU)/day vitamin D for adults increasing to 20 µg (800 IU)/day after age 71 years (24). Earlier Endocrine Society guidelines recommended 1500-2000IU/day of vitamin D for individuals at risk for osteoporosis (28). However, subsequent large trials found no skeletal benefit from supplementation (29, 30), prompting debate regarding optimal vitamin D levels (31). Current guidance does not recommend routine screening or specific intake targets (32). When recommending calcium or vitamin D supplements, there is a wide variety of choices that can accommodate patient preferences. For calcium, calcium carbonate is the least expensive salt and most concentrated. Calcium is better absorbed in divided doses of less than 500 mg. For those following a vegan eating pattern, Vitamin D_2_ can be recommended. Adequate vitamin D status facilitates calcium absorption, but this occurs well before current recommendations for vitamin D. Because vitamin D-mediated calcium absorption involves gene activation, which takes time, the 2 nutrients do not have to be coingested. Other micronutrients are needed for bone tissue maintenance and repair, but the evidence base is much less for the benefit of micronutrient supplements (33). The evidence for bioactive foods and ingredients remains limited and is largely focused on prevention or attenuation of bone loss rather than treatment of established osteoporosis. Polyphenol-rich foods such as prunes, soy, tea, and blueberries are among the more promising candidates, but optimal dosing, duration, and target populations remain to be established. A framework outlining the evidence needed to support health recommendations for bioactive foods has been published (33), and a similar framework for bioactive supplements is currently under review.

### Exercise and bone health

Exercise supports fracture prevention through 2 primary mechanisms: reducing falls and maintaining or improving bone mineral density. Accordingly, effective programs typically include balance or functional training (or Tai Chi) and resistance training, often combined with impact exercise.

#### Interventions targeting fall risk

There is high-certainty evidence that exercise can prevent falls (rate ratio 0.77, 95% confidence interval (CI) 0.71-0.83), 59 studies, 12 981 participants, and there is some evidence, albeit lower certainty, that exercise interventions may prevent fractures (risk ratio or RR 0.62; 95% CI 0.42-0.90) (34-37). When it comes to the types of exercise programs, analyses from systematic reviews suggest that exercise programs targeting training of balance, functional tasks (including gait), and coordination, sometimes combined with other types of exercise such as resistance training are effective for reducing falls, as is Tai Chi, whereas the evidence for other types of exercise, such as walking, dance, or resistance exercise alone, is less certain (36). Balance and functional training programs often target specific aspects of balance, such as anticipatory control, dynamic stability, functional stability limits, reactive control, and joint mobility (eg, ankles, hips) (38). Exercise program delivery by a healthcare professional (eg, physical therapist) may be more effective than programs delivered by individuals who are not healthcare or exercise professionals because they are tailored to be sufficiently challenging and address an individual's impairments or activity limitations (39). While physical activity guidelines often recommend balance exercise specifically for older adults (40), that may be because fall prevention is rarely studied in middle-aged adults, so there is no evidence on which to base guidelines. Because of the relevance of falls in fracture prevention, balance and functional training should be recommended to all adults at risk of fractures. In addition, fall risk assessment is associated with a reduction in fracture risk and should be performed alongside fracture risk assessment to further tailor interventions (35). Fall risk assessment algorithms have been proposed (41, 42), and start with basic fall risk screening questions (41).

#### Interventions targeting bone mineral density

Impact exercise, resistance exercise, or both in combination may maintain or increase BMD, and are thought to increase the forces on bone via ground reaction forces or muscle pull on bone (43-46). Weight-bearing or impact are terms often used interchangeably to describe exercise that is osteogenic but refer to a range of activities that may have variable efficacy. For example, walking or dancing are low-impact activities, whereas drop or depth jumps are high-impact activities. Walking is often recommended as weight-bearing or impact exercise, but in people with low bone mass, walking was reported to have no effect on total hip or femoral neck BMD, and a modest effect on spine BMD (mean difference 0.02 g/cm^2^, 95% CI [0.00, 0.03], 341 participants) (47).

Many trials that examine the effects of impact exercise on bone often combine it with other types of exercise, most often resistance training, which may be the best option if: (1) the goal is to maintain/increase BMD and (2) the individual can tolerate progressive impact exercise. In addition, it has been proposed that low-intensity resistance or impact exercise may not be enough of a stimulus, and moderate- or high-intensity exercise is effective for improving BMD (48, 49). There is evidence from meta-analyses of randomized controlled trials in postmenopausal women that resistance training (often with impact exercise) may improve BMD at the lumbar spine, hip and femoral neck (48). There are fewer trials in individuals with low bone mass, but one meta-analyses reported that resistance training may increase femoral neck (mean difference 0.02 g/cm^2^; 95% CI = 0.01-0.03; 521 participants, 5 studies) but not lumbar spine BMD or total hip BMD (44); there is a need for more trials of resistance and impact training in people with low bone mass to address the low precision in existing meta-analyses. To preserve both muscle and bone, one might recommend resistance training aligned with what is reported to be best for muscle strength outcomes in that the 2026 American College of Sports Medicine Position Stand on Resistance Training: resistance training using higher intensity loads (≥80% one-repetition maximum), through a complete range of motion, for 2-3 sets, at the beginning of training sessions (ie, resistance training first, not other types of training), and ≥2 sessions per week (50). Wearing weighted vests (during daily activities or exercise) does not improve bone strength when compared to control or exercise alone (51, 52). Research on the efficacy of Osteostrong is primarily observational, with inconsistent findings that are very low certainty, and evidence on safety is very limited (9).

**Figure 2:**
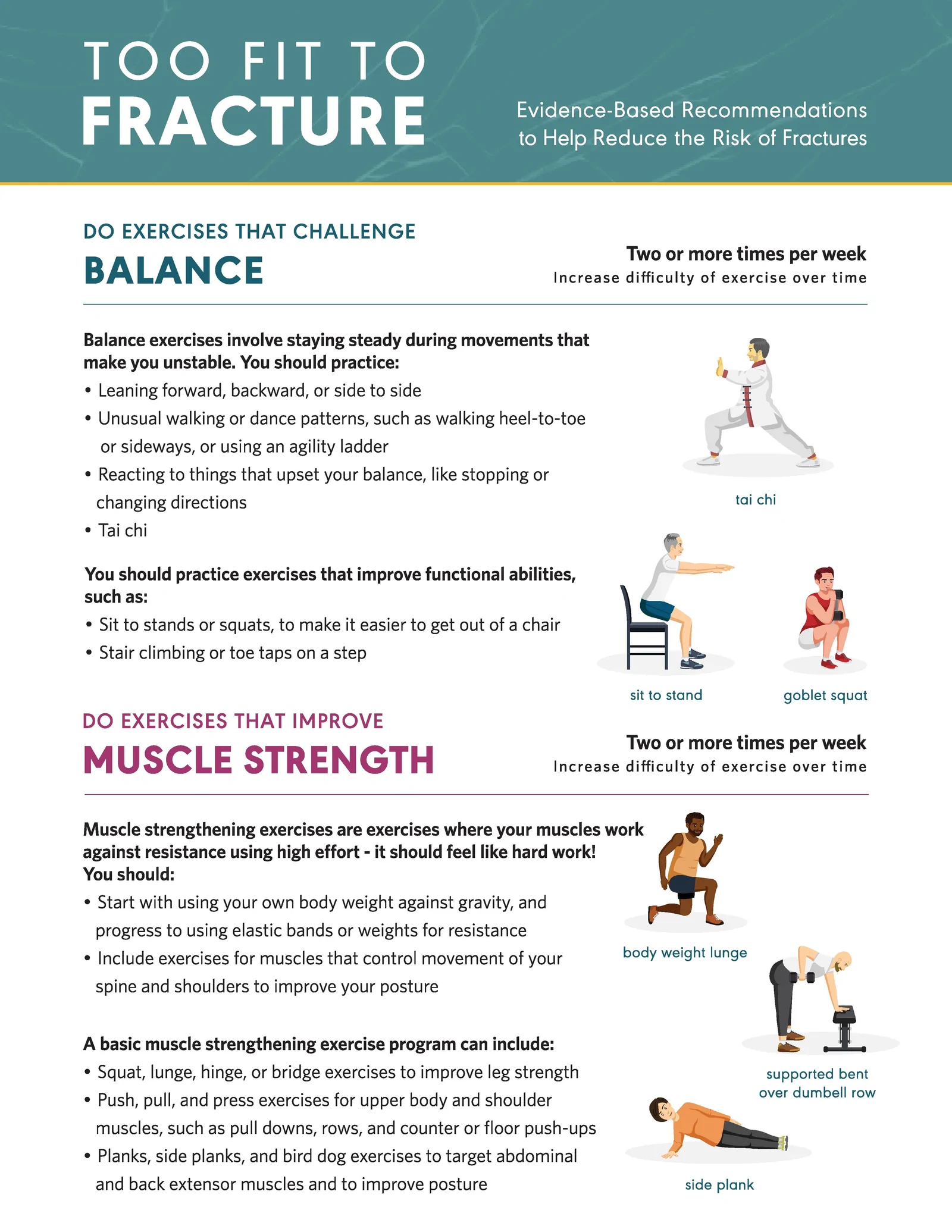

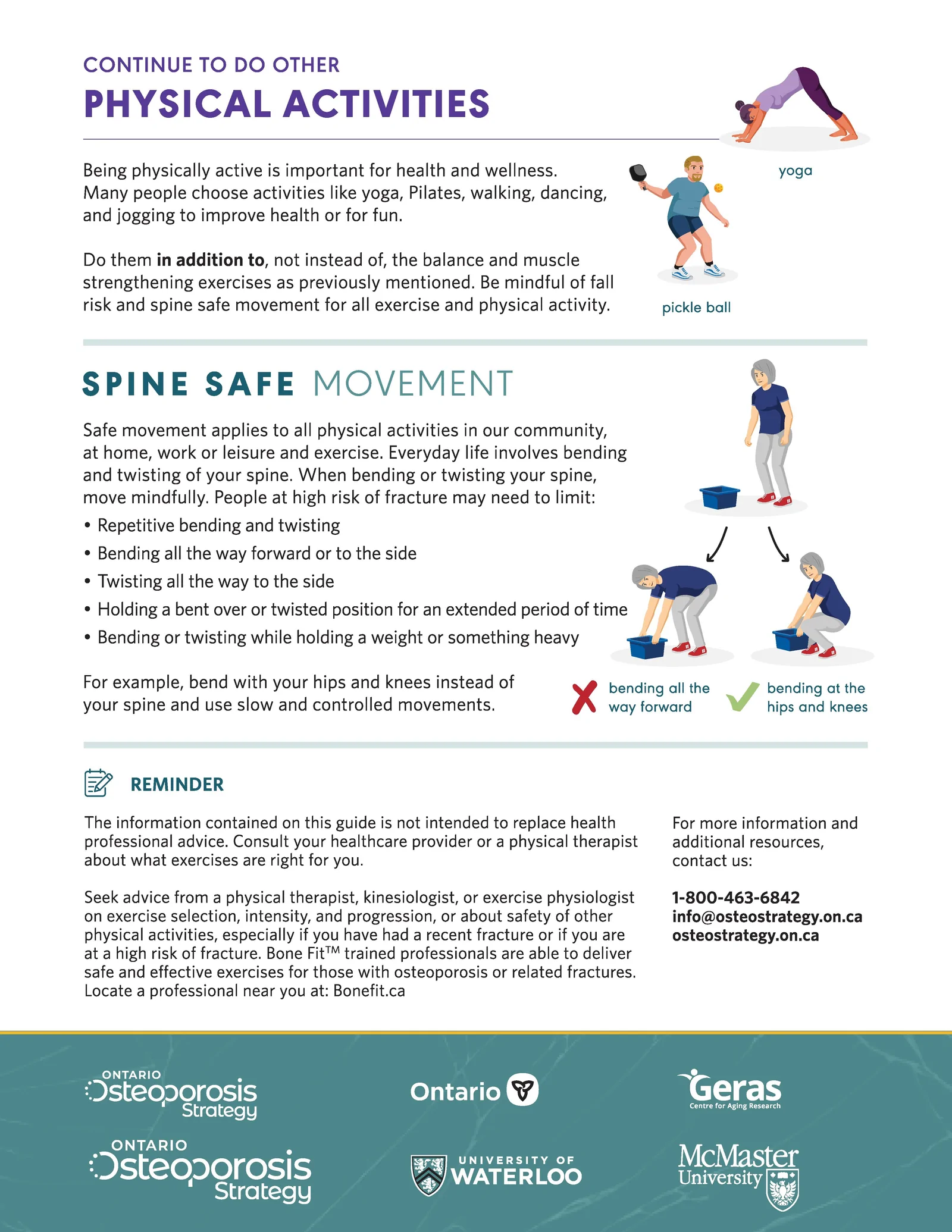
Evidence-based exercise recommendations to help reduce the risk of fractures. Reprinted with permission from Osteoporosis Canada.

Some clinicians or patients are concerned about exercise-related injuries, especially if patients are designing their own programs and performing them without guidance on form or tailoring to fall or fracture risk or physical functioning. Giving restrictive nonspecific advice (eg, don’t bend, don’t lift more than 5 lbs) creates fear and is a disincentive to physical activity. Instead, for high-risk individuals, discuss the types of movements that may need to be performed with caution eg, rapid, repetitive, weighted, sustained, or end-range lateral or forward flexion or twisting movements (53). Individuals should include, or progress to, moderate-impact (eg, hopping) or high-impact (eg, drop/depth jumps) exercise only if appropriate in the context of their fracture risk or other health conditions. For example, individuals with very high fracture risk (eg, history of hip or vertebral fracture) or certain health conditions may not be able to tolerate (or may not want to risk) doing impact exercise or heavy external loads; examples include spondyloarthropathy, arthritis in the lower extremities, pelvic organ prolapse, disc herniation, or stress incontinence. They could pursue alternative options that involve moderate-to-high muscle forces without impact or heavy external loads, such as power training, or strategic exercise selection where one increases difficulty without adding weight by altering position of the body relative to gravity, the number of limbs involved, or other factors. It is ideal if patients have access to or are willing to pay for exercise program design and instruction on form and progression by a certified exercise physiologist, strength and conditioning coach, or physical therapist with expertise in strength and impact training and osteoporosis. If they do not, they could consider lower-cost options if available, such as small group in-person training (which is lower cost than 1:1 training), reputable online resources (Fig. 2), or paying for a few sessions with an exercise physiologist to design a program they can follow independently. BoneFit™ is a continuing education program that trains exercise professionals in osteoporosis-specific exercise prescription, and patients may benefit from working with a BoneFit™-trained provider. Table 3 summarizes selected exercise resources clinicians can recommend. In general, the benefits of having a larger proportion of the population participating in resistance and balance training outweigh the risks, so clinicians should encourage participation and ensure that patients leave with a plan to start balance, strength, and, if appropriate, impact exercise that is aligned with their fall or fracture risk, preferences, and needs.

**Table 3:** Evidence-based patient education resources to support exercise and nutrition in osteoporosis

Resource	Format/access	Description and rationale
Osteoporosis Canada: Too Fit to Fracture^®^	Guideline-aligned landing page; videos https://osteoporosis.ca/too-fit-to-fracture/	Evidence-based exercise recommendations aligned with Canadian clinical practice guidelines, including education on strength, balance and functional exercises safely in people with osteoporosis.
Ontario Osteoporosis Strategy: Patient Resources	Downloadable booklet and handout on exercise and nutrition. https://osteostrategy.on.ca/patients/	Evidence-based patient education hub includes exercise and fall-prevention booklet, treatment information and practical self-management resources developed by multidisciplinary expert teams.
BoneFit™	Evidence-informed exercise training workshop for healthcare professionals and exercise practitioners, developed by Osteoporosis Canada. www.bonefit.ca and also available in the USA: https://www.bonehealthandosteoporosis.org/bonefit-usa/ Patients can explore whether there are BoneFit™ trained providers in their area.	Exercise professionals and healthcare professionals can attend online and in-person workshops to become “BoneFit™ trained”. The workshop is evidence-based and taught by experienced physical therapists and exercise physiologists.
Royal Osteoporosis Society (UK): Exercise and Physical Activity	Videos; downloadable fact sheets https://theros.org.uk/information-and-support/osteoporosis/living-with-osteoporosis/exercise-and-physical-activity-for-osteoporosis/	Structured videos demonstrating correct technique, frequency and progression of resistance, balance and posture exercises, with explicit guidance on spine-safe movement.
Dr. Lisa Moore, DPT—Brick House Bones	Type: YouTube channel and structured playlists Example videos: At-home and progressive workouts https://www.youtube.com/@DrLisaMooreDPT	Demonstrates resistance, balance, posture, and power training with detailed cues, regressions, and safety explanations, by a physical therapist, BoneFit™-trained; consistently references current evidence and guidelines
BonES Lab—boneslab.ca	Research-informed educational website https://uwaterloo.ca/bone-health-exercise-science-lab/	Website managed by researcher (Dr. Lora Giangregorio) that translates exercise and osteoporosis research into practical information for patients, with a blog and free newsletter.
BonES Lab—YouTube (@boneslab)	Short- and long-form videos https://www.youtube.com/@boneslab	YouTube Channel managed by researcher (Dr. Lora Giangregorio) with videos that translate osteoporosis research into practical information for patients, with emphasis on evidence appraisal and exercise science, including questions about Osteostrong, weighted vests, and other common questions.
Fragility Fracture Network (FFN): Vertebral Fragility Fracture Care Model and Patient Resources	Model of care framework; downloadable patient handouts and educational materials https://fragilityfracturenetwork.org/resources_list/vertebral-fragility-fracture-care-model/	Evidence-informed framework for the multidisciplinary management of vertebral fragility fractures across care settings. Handouts for patients on recovery expectations, early and later-phase spine-safe movement, exercise after a vertebral fracture, and nutritional considerations (eg, calcium, vitamin D, protein, weight management, constipation).
Onero Online (onero.online)	Subscription-based online program; instructional exercise videos; downloadable guidance https://onero.online/ Exercise professionals can become licensed to deliver the trademarked ONERO^®^ program https://onero.academy/	Onero Online is a lower-intensity, home-based exercise program developed to improve balance, mobility, muscle strength, and functional capacity in people with osteoporosis who cannot access supervised high-intensity training. Onero Online is distinct from the trademarked ONERO^®^ program and does not include the high-intensity resistance and impact exercises shown in randomized trials (eg, LIFTMOR) to increase bone mineral density. It was developed as a more accessible option for individuals who are early in their exercise journey or lack access to trained ONERO^®^ providers, with an explicit focus on reducing fracture risk via fall prevention rather than bone-targeted loading.
MelioGuide (melioguide.com)	Educational website; structured exercise plans; instructional videos; downloadable resources; books https://melioguide.com/	Educational platform created by a physical therapist with structured, progressive exercise programs and videos.
**Nutrition and Lifestyle Resources**		
International Osteoporosis Foundation (IOF): Nutrition resources	Educational web modules https://www.osteoporosis.foundation/health-professionals/prevention/nutrition	Detailed guidance on calcium, vitamin D, protein and other nutrients, including intake targets and practical dietary strategies linked to authoritative reference values.
Bone Health & Osteoporosis Foundation (BHOF)	Printable guides; handouts; infographics https://www.bonehealthandosteoporosis.org/patients	Practical patient tools to support implementation of bone-healthy eating patterns, calcium and vitamin D intake, and supplement decision-making.

### Pharmacologic treatment

**Figure 3:**
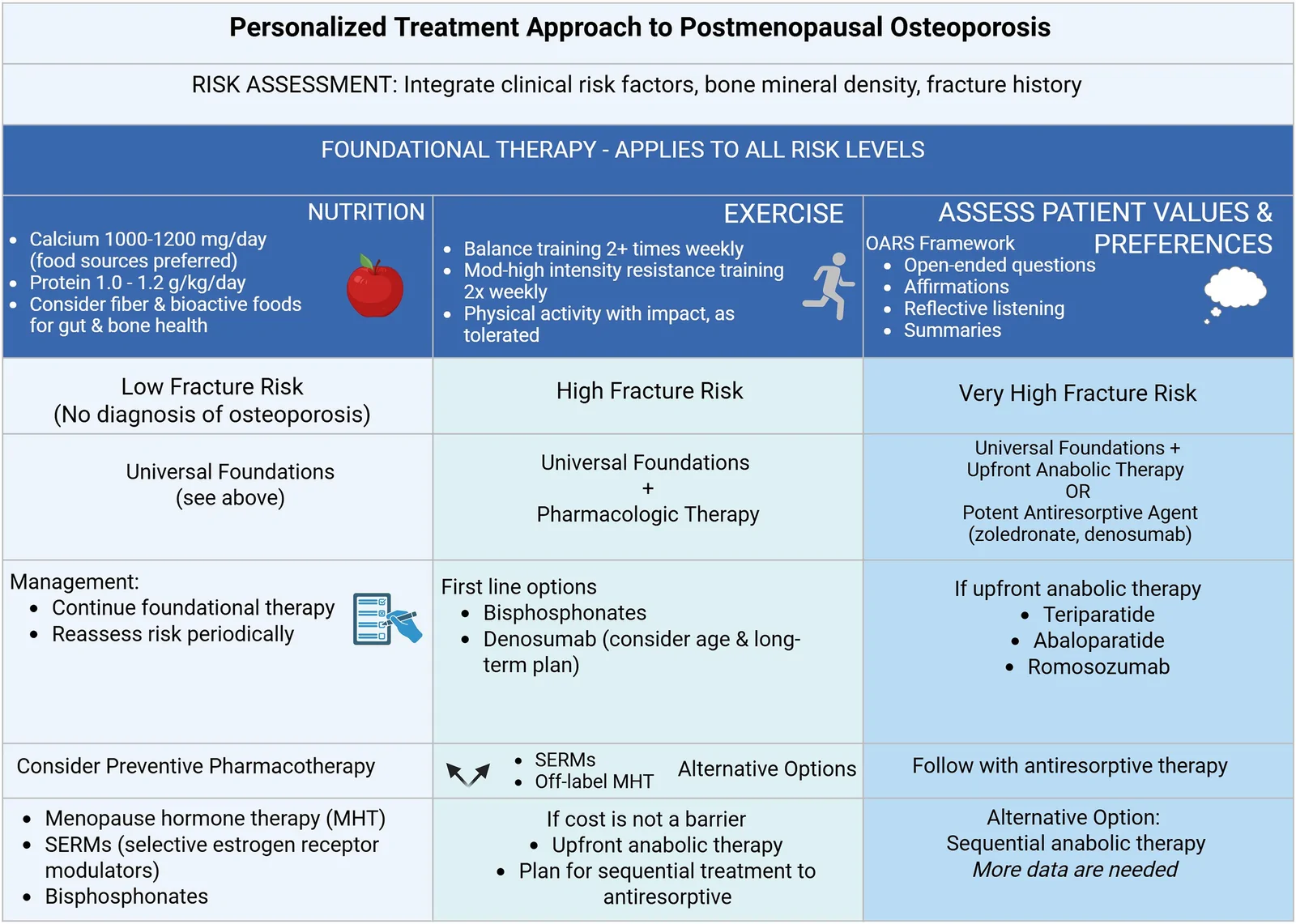
Personalized treatment approach to postmenopausal osteoporosis. Clinical risk assessment integrates fracture history, bone mineral density, and other risk factors to guide treatment selection. Foundational therapy—including nutrition, exercise, and patient-centered communication using shared decision-making principles—applies across all risk categories. For individuals at low fracture risk, management focuses on lifestyle optimization, periodic reassessment, and preventive pharmacotherapy when aligned with patient values and preferences. Patients at high fracture risk generally require antiresorptive therapy in addition to foundational measures, whereas those at very high fracture risk should be considered for upfront anabolic therapy or a potent antiresorptive agent. Treatment intensity and selection should be individualized and adjusted to align with patient goals and preferences. Sequential antiresorptive therapy is recommended following anabolic treatment to maintain skeletal benefits and reduce fracture risk.

The principles discussed above can be integrated into a personalized treatment framework that combines fracture risk assessment with evidence-based lifestyle interventions, pharmacologic therapy, and patient preferences. Figure 3 summarizes a practical approach to individualized osteoporosis management that can be adapted according to fracture risk, treatment goals, and patient values.

### Osteoporosis prevention

Aging and the hormonal changes of menopause are primary drivers of bone loss, yet despite the predictable decline in bone density across the menopausal transition (54), few women are offered pharmacologic therapy for osteoporosis prevention. The FDA has approved 3 classes of medications for prevention—estrogen, SERMs, and bisphosphonates—but they are infrequently prescribed for this purpose. More proactive strategies, including appropriate use of preventive medications, could meaningfully reduce the burden of osteoporosis and fractures among postmenopausal women.

### Treatment selection and sequence

The 2024 ASBMR/BHOF Task Force position statement on goal-directed osteoporosis therapy recommends selection of osteoporosis therapy based on an individual's fracture risk, with the overarching goal of achieving a T-score target > −2.5 within a reasonable time frame (55). Current guidelines stratify patients into high-risk and very-high-risk categories, with up-front anabolic therapy recommended for those at very high risk (56). Although there is no universally accepted definition of “very high fracture risk,” factors such as T-scores below −3.0, recent fractures, fractures while on approved osteoporosis medication, multiple fractures, fracture while taking medication known to cause skeletal harm, or very high FRAX scores (>30% major osteoporotic or > 4.5% hip fracture risk) are cited (57). In practice, however, treatment selection is shaped by far more than clinical risk alone; cost, insurance coverage, access to care, and patient values and preferences all influence the final therapeutic plan (58-60).

Unlike therapies for many other chronic diseases, most osteoporosis treatments are not typically continued indefinitely. It is therefore striking that age and life expectancy have not been incorporated into postmenopausal osteoporosis treatment guidelines. The ASBMR/BHOF position statement, for example, outlines treatment sequences expected to allow more than 50% of women to achieve a T-score above −2.5 within approximately 3 years (55). For a woman with a total hip T-score of −3.1 or a lumbar spine T-score of −3.7, the recommended sequence of goal-directed therapy is romosozumab followed by denosumab. Although this sequence was highly effective in the FRAME trial and associated with continued gains in bone mineral density (61), its long-term implications warrant consideration in younger women who may have a life expectancy of 30 years. We have a decade of safety data for denosumab (62), yet concerns persist about the implications of longer-term use, including the potential risk for jaw osteonecrosis and uncertainty about longer-term efficacy. An alternative approach for women with longer life expectancy may be to use sequential anabolic therapy, followed by a bisphosphonate, for those with very low baseline T-scores. Although evidence remains limited, repeat courses of teriparatide or romosozumab can be considered (abaloparatide is still capped at 24 months), with the ASBMR/BHOF position statement noting that a second anabolic course may be appropriate for individuals who remain at or return to very high fracture risk. A small study showed that repeat courses of romosozumab produced additional gains in bone density at the spine and hip when the interval therapy was a bisphosphonate or teriparatide; patients bridged with denosumab experienced gains at the spine only (63). These observations highlight the need for treatment algorithms that explicitly incorporate life expectancy and support more flexible, individualized sequencing across the multi-decade course of osteoporosis care.

One additional consideration for younger postmenopausal women is the potential role of menopause hormone therapy. Menopause hormone therapy is approved for prevention, but not for the treatment of osteoporosis. In the Women's Health Initiative, estrogen therapy was associated with significant reductions in hip, spine, wrist, and total fractures, with consistent effects observed in both the estrogen-alone and estrogen-plus-progestin arms, despite participants not being selected for low BMD or high fracture risk (64). Estrogen also produced approximately 3-5% increases in bone mineral density at the hip and spine over 3-6 years (65). Many postmenopausal women are diagnosed with osteoporosis before age 60, even though routine screening is not recommended until age 65. For these younger postmenopausal women, who have lower absolute fracture risk by virtue of age, menopause hormone therapy may represent a reasonable off-label treatment option in the absence of contraindications, with the added potential benefit of alleviating menopausal symptoms that are common in this age group (66). Selective estrogen receptor modulators (SERMs) are another option in this population. Raloxifene is FDA-approved for the treatment of osteoporosis, reducing vertebral fracture risk and lowering breast cancer risk in women at elevated risk (67). Bazedoxifene combined with conjugated equine estrogen is FDA-approved for osteoporosis prevention and the treatment of vasomotor symptoms (68); this combination does not increase mammographic breast density (69), and clinical trials evaluating its effects on other markers of breast cancer risk are ongoing (70).

### Limitations

Implementing comprehensive, patient-centered osteoporosis care is limited by time constraints, gaps in clinician training, limited multidisciplinary integration, and inadequate access to long-term lifestyle interventions. Patient-level barriers, including health literacy, socioeconomic factors, and time constraints, further complicate implementation. This manuscript focuses on postmenopausal osteoporosis and does not address osteoporosis in men or secondary osteoporosis. Future efforts should prioritize multidisciplinary training, practical clinical tools, and accessible care models to support personalized osteoporosis care.

### Returning to the cases

In the cases above, 2 women in their 50's presented with DXA-defined osteoporosis (T-scores <–3) without prior fractures. Neither met recommended calcium intake, and both were engaging in exercise approaches with limited evidence for skeletal benefit, despite strong motivation to improve bone health through lifestyle change. In Case 1, early surgical menopause, a major osteoporosis risk factor, had not prompted preventive pharmacotherapy. Given their very low BMD and a treatment goal of achieving a T-score >−2.5, up-front anabolic therapy was recommended in both cases.

In Case 1, the patient elected to proceed with anabolic therapy and achieved substantial gains in BMD. She also increased her dairy intake and transitioned from walking and hiking to resistance and impact training, sustaining a minor exercise-related injury that underscored the value of supervised instruction. Case 1 demonstrates substantial BMD gains with sequential anabolic therapy aligned with her goals.

In Case 2, the patient modified her long-term vegan diet to include lean meats after counseling regarding fracture risk and found support through a group exercise program, which helped reduce her medication hesitancy. Despite initial reluctance and insurance barriers, she ultimately initiated menopausal hormone therapy. Although estrogen alone was unlikely to achieve a target T-score >−2.5 given her very low hip BMD, meaningful gains in BMD were expected, and the approach aligned with her preferences.

Case 1's treatment selection and sequencing were perhaps more aggressive than current guidelines, while Case 2's approach was more conservative. Yet both women remained engaged in their treatment plans and remained fracture-free during follow-up—underscoring how individualized, preference-sensitive care can support positive outcomes across a range of therapeutic strategies.

In the current clinical environment, endocrinologists are increasingly expected to address a broad range of patient concerns, including questions about nutrition, exercise, and health information encountered online. This requires active listening, eliciting patient values and preferences alongside assessing medical risk, and translating this into personalized, comprehensive treatment plans that integrate lifestyle and pharmacologic therapies within a time-limited clinical setting. A proposed framework for integrating fracture risk assessment, foundational therapy, pharmacologic treatment, and patient preferences is shown in Fig. 3. By adopting this more comprehensive approach to bone health and by eliciting and responding to patient beliefs, values, and preferences, we can facilitate more effective treatment plans, improve patient engagement in osteoporosis care, and ultimately reduce fracture risk.

## Data Availability

Data sharing is not applicable to this article as no datasets were generated or analyzed during the current study.
